# Low HDL cholesterol is associated with elevated TNFR1 and TNFR2 levels in early diabetic kidney disease

**DOI:** 10.3389/fendo.2026.1716843

**Published:** 2026-01-23

**Authors:** Jianfang Su, Jing Su, Wei Wang, Jie Pan, Xianhui Zhang

**Affiliations:** 1 Department of Internal Medicine, Shanxi Children’s Hospital, Shanxi Maternal and Child Health Hospital, Taiyuan, China; 2 Department of Laboratory Medicine, The Fifth Clinical Medical College of Shanxi Medical University, Shanxi Provincial People’s Hospital, Taiyuan, China; 3 Department of Pathology, Stanford University School of Medicine, Palo Alto, CA, United States; 4 Department of Stem Cell Laboratory, Third Hospital of Shanxi Medical University, Shanxi Bethune Hospital, Taiyuan, China

**Keywords:** diabetic kidney disease, HDL cholesterol, inflammation, TNFR1, TNFR2, type 2 diabetes

## Abstract

**Background:**

Early diabetic kidney disease (DKD) remains difficult to diagnose accurately since microalbuminuria lacks sensitivity and specificity. Tumor necrosis factor receptors (TNFRs), especially TNFR1 and TNFR2, have emerged as potential markers of renal inflammation and injury. Dyslipidemia, particularly reduced high-density lipoprotein cholesterol (HDL), is associated with the inflammatory milieu in type 2 diabetes mellitus (T2DM).

**Objective:**

To investigate the association of serum TNFR1 and TNFR2 with early renal injury in T2DM and to determine the impact of HDL on TNFRs levels.

**Methods:**

A cross-sectional study was conducted in 258 T2DM patients (135 with normoalbuminuria, 123 with microalbuminuria) and 100 age- and sex-matched healthy controls. Serum TNFR1, TNFR2, lipid profile, fasting blood glucose (FBG), and estimated glomerular filtration rate (eGFR) were measured. Group differences were analyzed, and correlation and multivariable regression analyses were performed to identify determinants of TNFR levels.

**Results:**

Both TNFR1 and TNFR2 were significantly higher in patients with microalbuminuria compared with healthy controls (*P* < 0.001). TNFR1 levels increased progressively from healthy controls to normoalbuminuric and microalbuminuric groups, showing the strongest associations with UACR, eGFR, diabetes duration, and HDL. In multivariable regression, HDL emerged as the most significant negative predictor of both TNFR1 and TNFR2, independent of glycemic measures and other metabolic factors. Age was also independently associated with higher TNFR concentrations.

**Conclusion:**

Serum TNFR1 and TNFR2 are a sensitive biomarker for early renal injury in T2DM. Importantly, low HDL is independently associated with elevated TNFR1 levels, suggesting that lipid metabolism, beyond glucose control, plays a critical role in DKD progression. Monitoring HDL levels and targeting dyslipidemia may aid in the early prevention and intervention of DKD.

## 1 Introduction

Diabetic kidney disease (DKD) is one of the most common and serious microvascular complications of diabetes mellitus and represents the leading cause of end-stage renal disease worldwide (1). Early identification of individuals at high risk for DKD is crucial, as timely intervention can slow or even prevent the progression to advanced stages. However, conventional screening based on urinary albumin excretion and serum creatinine/eGFR has well-recognized limitations, as structural and functional renal abnormalities may precede overt albuminuria and creatinine-based decline (2).

Tumor necrosis factor-alpha (TNF-α) represents a pivotal pro-inflammatory cytokine in the pathogenesis of DKD. Its biological activities are primarily mediated by two distinct receptors, TNFR1 and TNFR2, which are shed from cell membranes into the systemic circulation as soluble forms. Clinical evidence has consistently shown that elevated serum concentrations of TNFR1 and TNFR2 are robust predictors of DKD development and progression, independent of conventional markers such as albuminuria and estimated glomerular filtration rate (eGFR) (3–6). Consequently, circulating TNFRs have emerged as highly sensitive biomarkers for detecting subclinical inflammation and early-stage renal injury. However, several critical knowledge gaps remain that hinder their clinical translation. First, the relative contributions of TNFR1 and TNFR2 during the early, albuminuria-negative stage of DKD are not well defined and may vary across populations, disease stages, and comorbid conditions. Second, the upstream metabolic and environmental factors regulating circulating TNFR levels are poorly understood. Elucidating these determinants is essential for accurate clinical interpretation of TNFR elevations and for guiding the development of targeted therapeutic interventions.

High-density lipoprotein cholesterol (HDL) is traditionally esteemed for its cardiovascular protective effects, largely attributed to its participation in reverse cholesterol transport as well as its potent anti-inflammatory and antioxidant activities (7). In the context of diabetes, diminished HDL-C levels are prevalent and closely associated with exacerbated systemic inflammation and endothelial dysfunction (8, 9). Emerging data further suggest that low HDL levels may potentiate renal damage by driving pro-inflammatory cascades and disrupting vascular homeostasis (10). However, the specific relationship between HDL and circulating TNFRs in the earliest stages of DKD remains to be fully elucidated.

In this study, we sought to examine circulating levels of TNFR1 and TNFR2 in patients with type 2 diabetes across different stages of renal involvement and to explore their relationship with HDL. We hypothesized that lower HDL levels would be associated with higher TNFR concentrations, reflecting heightened inflammatory activity in the early stages of DKD. Elucidating this relationship may provide new insights into the inflammatory mechanisms underlying DKD and help identify potential therapeutic targets for early intervention.

## 2 Materials and methods

### 2.1 Study design and participants

This cross-sectional study was conducted at Shanxi Provincial People’s Hospital (Shanxi, China) between April 2023 and October 2024. A total of 258 patients with type 2 diabetes mellitus (T2DM) were consecutively recruited and categorized into two groups based on their urinary albumin-to-creatinine ratio (UACR): the normoalbuminuria group (NA, n=135; UACR<30 mg/g) and the microalbuminuria group (MA, n=123; UACR 30–299 mg/g). In addition, 100 age- and sex-matched healthy individuals were enrolled as the healthy control group (HC).

The inclusion criteria for patients were as follows: a confirmed diagnosis of T2DM according to the American Diabetes Association criteria, age between 40 and 60 years, and stable glycemic control for at least three months prior to enrollment. Patients were excluded if they had acute diabetic complications, chronic kidney disease unrelated to diabetes, hepatic dysfunction, malignancies, autoimmune disorders, or were receiving glucocorticoids, immunosuppressive agents, or lipid-lowering medications that could influence HDL metabolism.

Demographic and clinical data, including age, sex, body mass index (BMI), disease duration, blood pressure, fasting blood glucose (FBG), lipid profiles, UACR, serum creatinine, and estimated glomerular filtration rate (eGFR), were collected from electronic medical records. UACR was measured at least twice within six months, and the mean value was used for analysis. The eGFR was calculated using the Modification of Diet in Renal Disease (MDRD) equation. The study protocol was reviewed and approved by the Ethics Committee of Shanxi Provincial People’s Hospital, and written informed consent was obtained from all participants before enrollment.

### 2.2 Measurements

Fasting blood samples were collected from all participants in the morning after an overnight fast of at least 10 hours. Samples were centrifuged at 3000 rpm for 10 minutes, and the serum was aliquoted and stored at −80 °C until further analysis. Serum TNFR1 and TNFR2 levels were measured using commercially available enzyme-linked immunosorbent assay (ELISA) kits (Wuhan Boster Biological Engineering, Wuhan, China). Each sample was measured in duplicate, and the mean value was used for statistical analysis. The intra-assay and inter-assay coefficients of variation for TNFR measurements were both <10%, indicating acceptable assay precision.

### 2.3 Statistical analyses

All statistical analyses were performed using SPSS software, version 26.0 (IBM Corp., Armonk, NY, USA). The normality of continuous variables was assessed using both the Shapiro-Wilk and Kolmogorov-Smirnov tests. Continuous variables with normal distributions were expressed as mean ± standard deviation (SD), whereas non-normally distributed variables were presented as median and interquartile range (IQR). Categorical variables were summarized as frequencies and percentages. Comparisons among the three groups were conducted using one-way analysis of variance (ANOVA) for normally distributed variables or the Kruskal-Wallis test for non-normally distributed variables. When significant differences were detected, *post hoc* pairwise comparisons with Bonferroni correction were performed. Categorical variables were compared using the chi-square test or Fisher’s exact test, as appropriate.

Pearson correlation coefficients were used to evaluate associations between serum TNFR1, TNFR2, and clinical variables, including HDL, FBG, UACR, and eGFR. For skewed data, log-transformation was applied before correlation analyses. Stepwise multiple linear regression analysis was then conducted to identify independent predictors of TNFR1 and TNFR2 levels in diabetic patients, including potential confounders such as age, disease duration, FBG, HDL, LDL, TC, TG, UACR, and eGFR. Results were expressed as unstandardized regression coefficients (B), standardized coefficients (Beta), and p-values, with a two-tailed *p* < 0.05 considered statistically significant. Multicollinearity was assessed using Variance Inflation Factors (VIF), with values < 5 considered acceptable.

## 3 Results

### 3.1 Baseline characteristics of study subjects

A total of 358 participants were enrolled in this study, including 100 healthy controls, 135 diabetic patients with NA, and 123 diabetic patients with MA. The baseline demographic and clinical characteristics of the study population are summarized in **Table 1**. There were no significant differences among the three groups in terms of sex distribution and age. Similarly, systolic and diastolic blood pressures did not differ significantly among the groups.

**Table 1:** Comparison of basic characteristics of study subjects.

Variables	HC (n=100)	NA group (n=135)	MA group(n=123)	*p*-value
Male (%)	55(55)	67(49.62)	62(50.40)	0.694
Age (years)	49.83 ± 5.08	50.23 ± 5.45	48.97 ± 6.59	0.335
Disease Duration (years)	–	8.8 ± 4.11	14.57 ± 6.19	<0.001*
Systolic Blood Pressure (mmHg)	123.34 ± 8.44	126.97 ± 7.33	127.26 ± 8.93	0.081
Diastolic Blood Pressure (mmHg)	74.13 ± 8.69	73.26 ± 7.51	73.51 ± 6.64	0.882
Fasting Blood Glucose (mmol/L)	4.78 ± 2.86	7.1(5.87-7.56)	7.33 ± 2.86	<0.001*
Triglycerides (TG, mmol/L)	1.05(0.84-1.39)	1.17(1.05-2.15)	1.42(1.25-1.94)	0.742
Total Cholesterol (TC, mmol/L)	4.16 ± 0.57	4.32 ± 0.56	4.44 ± 0.54	0.051
LDL (mmol/L)	2.48 ± 0.59	2.49 ± 0.68	2.70 ± 0.81	0.333
HDL (mmol/L)	1.03(0.86-1.22)	0.92(0.76-1.13)	0.81(0.66-0.98)	0.007*
UACR (mg/g)	5.47(2.93-8.72)	13.32(9.97-19.52)	140.74(131.89-166.43)	<0.001*
Serum Creatinine (μmol/L)	52.31 ± 6.16	59.79 ± 7.18	66.78 ± 10.38	<0.001*
eGFR (ml/min/1.73m²)	126.20 ± 8.15	127.49 ± 15.52	109.50(101.90-130.38)	<0.001*

Data are given as mean ± SD, median (IQR) or n (%). *indicate statistically significant *p* < 0.05. UACR, Urinary Albumin-to-Creatinine Ratio.

As expected, the duration of diabetes was significantly longer in the MA group compared with the NA group (*p* < 0.001). FBG levels were significantly higher in both diabetic groups compared with healthy controls. With respect to lipid profiles, HDL levels decreased progressively across the groups, with the lowest levels observed in the MA group (*p* = 0.007). No significant differences were observed for TC, TG, or LDL cholesterol among the three groups. Consistent with the grouping criterion, UACR increased stepwise from HC to NA, with a marked elevation in the MA group (*p* < 0.001). Reflecting a decline in renal function, the MA group exhibited significantly higher serum creatinine levels and lower eGFR compared with both the NA and HC groups (*p* < 0.001).

### 3.2 Comparison of serum TNFR1 and TNFR2 levels between groups

**Figure 1:**
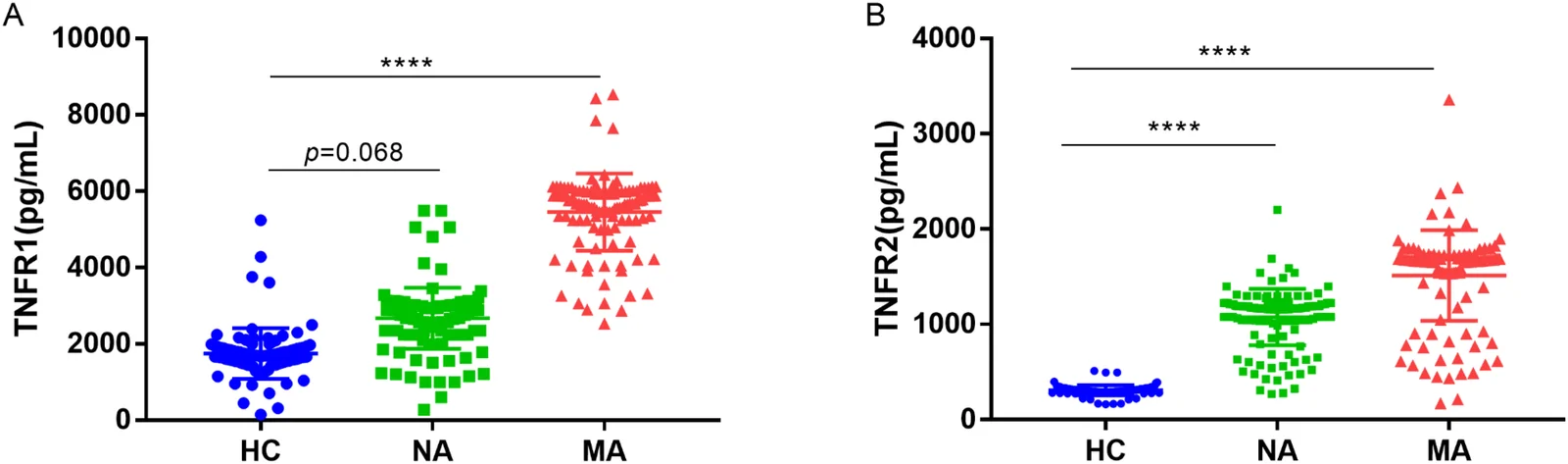
Comparison of serum TNFR1 and TNFR2 levels across study groups. Serum concentrations of **(A)** TNFR1 and **(B)** TNFR2 were measured by ELISA in HC (n=100), T2DM patients with NA (n=135), and T2DM patients with MA (n=123). Individual data points are shown, with horizontal lines representing the mean ± SD. Differences among the three groups were analyzed using the Kruskal-Wallis test, followed by *post hoc* pairwise comparisons with Bonferroni correction. In subplot A, while TNFR1 levels showed an increasing trend in the NA group, the difference compared to HC was not statistically significant (*p* = 0.068). In subplot B, TNFR2 was significantly elevated even in the NA stage compared to HC. HC, healthy controls; NA, normoalbuminuria; MA, microalbuminuria; TNFR1, tumor necrosis factor receptor 1; TNFR2, tumor necrosis factor receptor 2. Significance: **** *p* < 0.001.

As shown in **Figure 1**, serum levels of both TNFR1 and TNFR2 were significantly elevated in patients with T2DM, particularly in those with MA, compared with HC *(p* < 0.001). Although the difference in TNFR1 levels between the NA and HC groups did not reach statistical significance (*p* = 0.068), a clear trend toward higher levels was evident in the NA group. Among the diabetic participants, the MA group exhibited the highest concentrations of both TNFR1 and TNFR2. Notably, the progression from NA to MA was marked by a more pronounced increase in TNFR1 (2.04-fold) compared to the further increase in TNFR2 (1.41-fold), suggesting that TNFR1 may be a more dynamic indicator of advancing renal injury once albuminuria begins to manifest.

### 3.3 Correlation analysis between TNFRs and metabolic risk factors in diabetic patients

Correlation analyses were conducted to evaluate the relationships between circulating TNFR1 and TNFR2 levels and metabolic risk factors in the diabetic cohort (**Table 2**). Both TNFR1 and TNFR2 were positively correlated with age and diabetes duration. Similarly, FBG exhibited a significant positive correlation with both receptors.

**Table 2:** Correlation analysis of TNFR1 and TNFR2 with metabolic risk factors in the DM subjects.

Variables	TNFR1		TNFR2	
	r-value	*p*-value	r-value	*p*-value
Age (years)	0.376	<0.001*	0.556	<0.001*
Disease Duration (years)	0.537	<0.001*	0.407	<0.001*
Fasting Blood Glucose (mmol/L)	0.564	<0.001*	0.572	<0.001*
Total Cholesterol (TC, mmol/L)	-0.122	0.313	-0.135	0.265
LDL (mmol/L)	-0.008	0.7	-0.218	0.069
HDL (mmol/L)	-0.459	<0.001*	-0.470	<0.001*
UACR (mg/g)	0.667	<0.001*	0.368	0.002*
Serum Creatinine (μmol/L)	-0.003	0.979	-0.075	0.537
eGFR (ml/min/1.73m²)	-0.068	0.575	-0.013	0.916

*indicate statistically significant *p* < 0.05.

In terms of renal parameters, UACR showed a strong positive correlation with TNFR1 (*r* = 0.667, *p* < 0.001) and a moderate correlation with TNFR2 (*r* = 0.368, *p* = 0.002). In contrast, neither serum creatinine nor eGFR correlated significantly with either receptor. Notably, HDL was inversely correlated with both TNFR1 and TNFR2 (*p < 0.001*). No significant associations were observed between the TNFRs and TC or LDL cholesterol. These results indicate that elevated TNFR1 and TNFR2 levels are linked to poorer glycemic control, longer diabetes duration, reduced HDL-C, and higher urinary albumin excretion. Moreover, TNFR1 exhibited a stronger correlation with UACR than TNFR2, suggesting a potentially greater role in early diabetic kidney injury.

### 3.4 Multivariable regression analysis

To identify independent factors influencing circulating TNFR1 and TNFR2, multivariable linear regression analysis were performed, adjusting for age, disease duration, FBG, UACR, and HDL (**Table 3**). For TNFR1, the analysis identified age, UACR, and HDL as independent predictors. UACR exhibited the strongest positive association, followed by age, while HDL was inversely correlated with TNFR1 levels. For TNFR2, age and HDL remained significantly associated, whereas UACR showed a trend toward association that did not reach statistical significance (*p* = 0.052). Disease duration and FBG were not independently associated with either receptor in the fully adjusted model. These results suggest that HDL is a robust negative determinant of both TNFR1 and TNFR2, independent of key metabolic and renal covariates, highlighting its potential role in modulating inflammatory activation in early diabetic kidney disease.

**Table 3:** Multivariable regression analysis of TNFR1 and TNFR2 with metabolic risk factors in the DM subjects.

	Variables	Unstandardized coefficients (B)	Std. error	Standardized coefficients (Beta)	t	*p*-value
TNFR1	Constant	-2821.164	1364.346	–	-2.068	0.043
	Age	108.914	24.187	0.343	4.503	<0.001*
	Disease Duration	26.025	26.261	0.081	0.991	0.325
	Fasting Blood Glucose	180.833	108.657	0.14	1.664	0.101
	UACR	14.737	2.27	0.529	6.492	<0.001*
	HDL	-1862.255	552.593	-0.261	-3.37	0.001*
TNFR2	Constant	-1753.246	835.146	–	-2.099	0.04
	Age	57.3	14.805	0.413	3.87	<0.001*
	Disease Duration	5.744	16.075	0.041	0.357	0.722
	Fasting Blood Glucose	61.086	66.511	0.108	0.918	0.362
	UACR	2.755	1.39	0.226	1.982	0.052
	HDL	-843.32	338.254	-0.271	-2.493	0.015*

* indicate statistically significant *p* < 0.05.

## 4 Discussion

In this study, we found that circulating TNFR1 and TNFR2 levels were significantly elevated in patients with T2DM, including those without overt albuminuria. Notably, TNFR1 exhibited the strongest association with established markers of renal injury, particularly UACR and eGFR, indicating that TNFR1 may serve as a more sensitive indicator of early diabetic kidney injury. These findings are consistent with previous longitudinal studies demonstrating that higher baseline levels of TNFR1 and TNFR2 predict accelerated eGFR decline and an increased risk of progression to advanced CKD and end-stage renal disease, independent of baseline albuminuria and traditional clinical risk factors (11, 12).

Given that albuminuria alone does not fully reflect early renal injury, particularly during the initial stages of DKD, incorporating TNFR measurements may enhance the detection of subclinical inflammation and kidney damage at a point when therapeutic interventions could be more effective. In our cohort, TNFR1 demonstrated a stronger correlation with both UACR and eGFR compared to TNFR2, suggesting potential biological differences between the two receptors.

TNFR1 is primarily involved in pro-inflammatory and pro-apoptotic signaling, whereas TNFR2 has been implicated in tissue repair, cell survival, and immune regulation. This functional divergence indicates that TNFR1 may be more directly associated with structural kidney injury, while TNFR2 could reflect compensatory or modulatory responses to ongoing damage (3). The predominance of TNFR1 observed in our findings may be related to the characteristics of our study population, which included Chinese patients with T2DM at an early stage of DKD, or to differences in receptor shedding dynamics and clearance rates. Further mechanistic studies are needed to elucidate the precise cellular origins and biological pathways contributing to circulating TNFRs.

It is also noteworthy that while numerous cohorts have demonstrated the predictive value of TNFRs for DKD progression, the magnitude and receptor-specific effects are not entirely consistent across studies. Some investigations have reported a stronger association with TNFR2 or observed weaker predictive performance overall (13). These discrepancies may be attributable to differences in ethnicity, comorbidities, study design, or analytical techniques. Understanding these sources of variation will be essential for standardizing the clinical application of TNFRs as biomarkers for early DKD detection and risk stratification. The clinical relevance of TNFRs extends beyond their predictive value to their responsiveness to therapeutic interventions. A recent prospective study by Otoda et al. demonstrated that a reduction in albuminuria following 4 months of SGLT2 inhibitor treatment was independently associated with a decrease in serum TNFR1 levels (14). These findings further support the potential of TNFRs as dynamic clinical indicators for assessing treatment response and monitoring renal inflammatory activity.

Furthermore, a novel and clinically significant finding of our study is that HDL emerged as an independent and robust negative predictor of circulating TNFR levels. This observation suggests that reduced HDL may is linked to activation of the TNF signaling pathway, thereby reflecting renal inflammation even before the clinical manifestation of albuminuria. Although the cross-sectional nature of our study precludes causal inference, based on prior mechanistic literature, we hypothesize a potential causal pathway where reduced HDL functional reserve facilitates renal inflammation. There are biologically plausible mechanisms to support this hypothesis. In addition to its central role in reverse cholesterol transport, HDL possesses potent anti-inflammatory and antioxidant properties. Experimental studies have demonstrated that HDL particles and their associated proteins and lipids can suppress macrophage activation, attenuate Toll-like receptor (TLR)-mediated cytokine production via ATF3-dependent transcriptional repression, and protect endothelial cells from oxidative stress and inflammatory injury (7, 15). Consequently, a reduction in circulating HDL levels may result in the loss of these protective functions, leading to unrestrained inflammatory signaling, enhanced activation of the TNF pathway, and increased shedding of TNFRs into the circulation.

It is also important to recognize that HDL quantity alone does not fully capture its cardiometabolic or renoprotective functions, as HDL particles undergo qualitative alterations in metabolic disorders and CKD. Recent reviews and lipidomic studies have documented substantial compositional and functional changes in HDL in CKD, including the loss of anti-inflammatory and antioxidative properties, which can diminish HDL’s capacity to suppress cytokine signaling and may partly explain the heterogeneity observed in epidemiologic associations (16, 17). Contemporary epidemiologic studies of HDL and renal or cardiovascular risk have reported complex, sometimes non-linear relationships, including U-shaped associations, underscoring the importance of considering particle functionality, extreme HDL phenotypes, and population-specific context (10, 18). Accordingly, the inverse association observed in our study between HDL and circulating TNFRs may, at least in part, reflect that lower HDL levels serve as a surrogate for reduced HDL functional reserve. Future studies assessing HDL functionality, such as cholesterol efflux capacity, anti-inflammatory activity, and proteomic/lipidomic composition, will be critical to clarify causal links between HDL dysfunction and TNFR-mediated inflammatory activation in early DKD.

Interestingly, our regression analysis also revealed that age was independently associated with elevated circulating TNFR levels. Age-related immune dysregulation, commonly referred to as “inflammaging,” is characterized by chronic, low-grade inflammation and increased production of pro-inflammatory cytokines, including TNF-α (19–21). With advancing age, both innate and adaptive immune systems undergo functional decline, resulting in a systemic shift toward a pro-inflammatory state that may accelerate renal microvascular injury (22). Additionally, aging is associated with alterations in lipid metabolism, including qualitative changes in HDL particles that reduce their protective anti-inflammatory and antioxidative functions (23). Consequently, age may influence circulating TNFR levels through two complementary mechanisms: direct enhancement of TNF pathway activation via immune senescence and indirect modulation through impaired HDL-C functionality. These interrelated mechanisms likely contribute to the higher TNFR concentrations observed in older participants in our study, even after adjustment for traditional metabolic and renal risk factors.

Our findings have several important implications for clinical practice and future research. First, TNFR1 and TNFR2 may serve as sensitive early biomarkers for detecting subclinical renal inflammation in T2DM patients. These markers could complement traditional measures, such as UACR, potentially improving risk stratification and enabling earlier intervention. Second, the observed inverse association between HDL and TNFRs suggests that strategies aimed at preserving or improving HDL quantity and functionality may help attenuate inflammatory activation and slow the progression of DKD. Third, the independent association of age with circulating TNFR levels highlights the influence of age-related immune changes on inflammatory biomarkers. This finding underscores the need for earlier screening and more intensive monitoring of subclinical kidney injury in older adults with diabetes, even in the absence of elevated UACR.

Despite these strengths, several limitations should be acknowledged. First, the cross-sectional design precludes causal inference, and we were unable to directly assess HDL functionality or measure upstream inflammatory mediators such as TNF-α. Second, we lacked detailed data on baseline medications like ACE inhibitors and SGLT2 inhibitors. This may introduce confounding bias, as these drugs significantly modulate the inflammatory and renal pathways investigated here. Third, the ethnic and clinical characteristics of our cohort may differ from those of other populations, potentially influencing the observed associations. Future studies should incorporate longitudinal follow-up to evaluate whether baseline HDL-C, TNFR levels, and age-related factors predict eGFR decline and hard renal outcomes. Mechanistic studies are also warranted to elucidate the cellular sources of circulating TNFRs and to investigate the potential modulatory effects of medications, including statins, fibrates, and SGLT2 inhibitors, on the HDL-TNF axis.

In conclusion, our findings support a model in which early activation of the TNF pathway is involved in DKD pathogenesis, with HDL serving as a key negative regulator and age acting as an independent driver of inflammatory activation. Targeting the HDL-inflammation-TNFR axis, while accounting for age-related immune alterations, may offer a promising strategy for the early identification and prevention of DKD. Prospective studies integrating biomarker assessment, mechanistic investigation, and interventional trials are needed to validate and extend these observations.

## Data Availability

The raw data supporting the conclusions of this article will be made available by the authors, without undue reservation.
