# Correction: Low HDL cholesterol is associated with elevated TNFR1 and TNFR2 levels in early diabetic kidney disease

**DOI:** 10.3389/fendo.2026.2024429

**Published:** 2026-10-23

**Authors:** Jianfang Su, Jing Su, Wei Wang, Jie Pan, Xianhui Zhang

**Affiliations:** 1 Department of Internal Medicine, Shanxi Children’s Hospital, Shanxi Maternal and Child Health Hospital, Taiyuan, China; 2 Department of Laboratory Medicine, The Fifth Clinical Medical College of Shanxi Medical University, Shanxi Provincial People’s Hospital, Taiyuan, China; 3 Department of Pathology, Stanford University School of Medicine, Palo Alto, CA, United States; 4 Department of Stem Cell Laboratory, Third Hospital of Shanxi Medical University, Shanxi Bethune Hospital, Taiyuan, China

**Keywords:** diabetic kidney disease, HDL cholesterol, inflammation, TNFR1, TNFR2, type 2 diabetes

In the published article, there was an error in the order of affiliation superscripts for the corresponding author “Xianhui Zhang”. Instead of “Xianhui Zhang¹,^4^*”, it should be “Xianhui Zhang^4^,¹*”. Affiliation ^4^ represents the primary institutional affiliation, while affiliation ¹ is the secondary affiliation. The full text of all affiliation descriptions remains unchanged.

The original article has been updated.

